# Dissociable Effects of Reward on P300 and EEG Spectra Under Conditions of High vs. Low Vigilance During a Selective Visual Attention Task

**DOI:** 10.3389/fnhum.2020.00207

**Published:** 2020-06-24

**Authors:** Jia Liu, Chi Zhang, Yongjie Zhu, Yunmeng Liu, Hongjin Sun, Tapani Ristaniemi, Fengyu Cong, Tiina Parviainen

**Affiliations:** ^1^School of Biomedical Engineering, Dalian University of Technology, Dalian, China; ^2^Faculty of Information Technology, University of Jyväskylä, Jyväskylä, Finland; ^3^Department of Psychology, Neuroscience and Behaviour, McMaster University, Hamilton, ON, Canada; ^4^School of Artificial Intelligence, Faculty of Electronic Information and Electrical Engineering, Dalian University of Technology, Dalian, China; ^5^Key Laboratory of Integrated Circuit and Biomedical Electronic System, Dalian University of Technology, Dalian, China; ^6^Centre for Interdisciplinary Brain Research, Department of Psychology, Faculty of Education and Psychology, University of Jyvaskyla, Jyvaskyla, Finland

**Keywords:** vigilance, mental fatigue, motivation, selective visual attention, event-related potential, event-related spectral perturbation

## Abstract

The influence of motivation on selective visual attention in states of high vs. low vigilance is poorly understood. To explore the possible differences in the influence of motivation on behavioral performance and neural activity in high and low vigilance levels, we conducted a prolonged 2 h 20 min flanker task and provided monetary rewards during the 20- to 40- and 100- to 120-min intervals of task performance. Both the behavioral and electrophysiological measures were modulated by prolonged task engagement. Moreover, the effect of reward was different in high vs. low vigilance states. The monetary reward increased accuracy and decreased the reaction time (RT) and number of omitted responses in the low but not in the high vigilance state. The fatigue-related decrease in P300 amplitude recovered to its level in the high vigilance state by manipulating motivation, whereas the fatigue-related increase in P300 latency was not modulated by reward. Additionally, the fatigue-related increase in event-related spectral power at 1–4 Hz was sensitive to vigilance decrement and reward. However, the spectral power at 4–8 Hz was only affected by the decrease in vigilance. These electrophysiological measures were not influenced by motivation in the state of high vigilance. Our results suggest that neural processing capacity, but not the timing of processing, is sensitive to motivation. These findings also imply that the fatigue-related impairments in behavioral performance and neural activity underlying selective visual attention only partly recover after manipulating motivation. Furthermore, our results provide evidence for the dissociable neural mechanisms underlying the fatigue-related decrease vs. reward-related increase in attentional resources.

## Highlights

- Time-on-task impairs performance in a selective visual attention task.
- Lower vigilance is associated with decreased amplitude and increased latency of P300.
- Monetary rewards improve P300 amplitude, but not P300 latency.
- The event-related spectral power at 1–4 Hz is sensitive to vigilance decrement and reward, whereas the spectral power at 4–8 Hz is sensitive only to vigilance decrement.
- Reward improves P300 amplitude and spectral power at 1–4 Hz only in the low and not in the high vigilance state.

## Introduction

Although we are subjected to constant visual information in daily life, our visual capacity to process this information is limited. To perform in an efficient and goal-directed manner, we need to continuously distinguish the relevant information from the visual environment and allocate our limited attentional capacity to the selected target objects, a phenomenon referred to as selective visual attention (Moore and Zirnsak, 2017). As outlined by Robert and Duncan (1995), selective visual attention is characterized by two basic phenomena: the ability to filter out task-irrelevant stimuli and the limited capacity for task-relevant information processing, both of which leading to reduced accuracy when the target number increases. We experience selective visual attention in many daily activities. For example, customers find the target objects among colorful irrelevant sales; car drivers filter out irrelevant surroundings and detect the relevant road marks and traffic lights. However, prolonged engagement in selective attention tasks inevitably leads to increased errors, deactivated performance goals, diminished motivation to continue performing the task (Boksem et al., 2005), and an increase in mental fatigue (Kok, 2001; Lal and Craig, 2001; Gergelyfi et al., 2015; Benoit et al., 2019).

Mental fatigue is caused by prolonged cognitive task performance (Gergelyfi et al., 2015). It is considered a related concept but distinct from arousal, which often refers to a physiological state and is closely linked with the transition between wakefulness and sleep (Shen et al., 2006). Mental fatigue is a cumulative process, accompanied by a feeling of indolence, reduced motivation, and impaired performance (Lal and Craig, 2001). What is more, mental fatigue exhibits more cognitive elements than arousal. Based on different causal factors, two types of mental fatigue can be identified: sleep- and task-related (May and Baldwin, 2009). The former results from accumulated sleep debt, whereas the latter from prolonged task engagement (May and Baldwin, 2009). In the present study, we aimed to examine the task-related mental fatigue, specifically related to attentional resources. The attention-requiring task performance over a prolonged duration pointedly refers to vigilance decrement (Mackworth et al., 1964), which is likely identical or very closely related to mental fatigue (Oken et al., 2006). For this reason, both terms have been used interchangeably in previous studies (Taya et al., 2018; Reteig et al., 2019).

Vigilance decrement has been reported as a major factor in a large proportion of road crashes due to the reduction of attentional resources. Although the risks of vigilance decrement have received much attention, the underlying neurophysiological mechanisms have not yet been established (Lorist et al., 2005; Tops et al., 2006; Benoit et al., 2019). In earlier research, three core concepts around vigilance decrement or mental fatigue have emerged, namely, active fatigue, passive fatigue, and motivational control. Active fatigue is a result of an excessive workload—needed to carry out a task over a prolonged duration, resulting in the depletion of cognitive resources (Helton and Warm, 2008). Passive fatigue is a result of a lower workload—needed to engage in prolonged, but relatively easy tasks (May and Baldwin, 2009). Motivational control plays an important role in vigilance decrement, as it reflects the level of willingness to perform a task. Motivational control is linked with the process of subconscious balancing between costs and benefits to expend or conserve energy (Kurzban et al., 2013a). For instance, Kurzban and colleagues suggested that people experienced performance reductions over time when the costs outweighed the benefits (Kurzban et al., 2013b). Recent studies recognize that these three core concepts are not mutually exclusive, and there are still limitations in the core concepts account for changes induced by fatigue (Boksem and Tops, 2008; Seli et al., 2015; Thomson et al., 2015). Therefore, the hybrid models synthesizing different concepts have emerged to complement the limitations. For example, Boksem and Tops (2008) proposed a framework of mental fatigue that integrated the motivational control and energetical costs, suggesting that people would no longer maintain their performance when the energetical resources depleted, although the costs outweighed the benefits. All in all, it is still unclear why task performance deteriorates with time-on-task.

The influence of motivation on prolonged task performance has been studied by subsequently providing monetary rewards. The effects on response selection (Möckel et al., 2015), action monitoring (Boksem et al., 2006), and sustained attention (Reteig et al., 2019) have been previously shown. Although numerous studies have demonstrated that monetary rewards can improve performance when provided after long-term tasks (Lorist et al., 2005; Boksem et al., 2006; Hopstaken et al., 2015), the neural mechanisms upon which this improvement builds on are not established. Moreover, the effect of reward on performance in different (i.e., high vs. low) vigilance states has rarely been approached.

**Figure 1:**
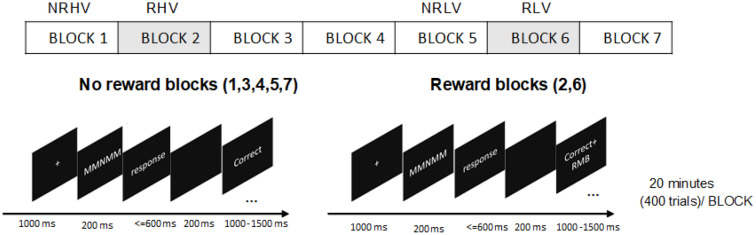
The procedure of experiment design and the trial structure of flanker task.

To explore the effects of motivation on behavioral performance and brain electrophysiology in high and low vigilance states, we conducted a 140-min selective visual attention task and provided monetary rewards for successful task performance in the early stage (during the 20- to 40-min interval) and in the late stage (during the 100- to 120-min interval; Figure 1). By utilizing brain electrophysiological measures derived from high-temporal-resolution electroencephalograms (EEGs), we focused on time domain [event-related potential (ERP) P300 amplitude and latency] and time-frequency domain [event-related spectral perturbations (ERSPs)] variables as electrophysiological markers of visually induced neural activations. We further quantified the degree of recovery of behavioral and electrophysiological measures in the low vigilance state after motivation manipulation.

The stimulus-locked ERP component P300 has received much attention as a potential indicator of mental workload in a selective visual attention task (Faber et al., 2012). The amplitude of P300 was proved to be a useful measure of processing capacity that correlates positively with the accuracy of the memory search task (Kok, 2001). Furthermore, the latency of P300 was suggested to be an indicator of mental chronometry as demonstrated by its positive correlation with reaction time (RT; Verleger, 1997). While reports about the effect of time-on-task on the P300 component are diverse, the study of Faber et al. (2012) did not find a significant decrease in the P3b amplitude during prolonged engagement in a selective visual attention task. Boksem et al. (2006) also showed that the P300 amplitude did not change with time-on-task, but the P300 latency increased with vigilance decrement. Although the P300 amplitude and latency have been widely used in studies on vigilance (Kato et al., 2009; Käthner et al., 2014; Hopstaken et al., 2015), most results are limited to conventional ERP analysis.

It is also valuable to explore how the oscillatory dynamics reflect changes in attentional allocation and information processing during a selective visual attention task. Frontal theta oscillations have been shown to be related to the allocation of attention to task-relevant visual and auditory stimuli (Keller et al., 2017). Oscillations in the delta band have been implicated in attention and salience detection and are associated with vigilance levels and motivation (Knyazev, 2012). It has also been suggested that EEG delta oscillations are an indicator of attention to internal processing during the performance of mental tasks (Harmony et al., 1996). Compared with traditional time- and phase-locked ERP analysis, the changes in spectral power provided by two-dimensional time-frequency analysis could provide a better account of the neural mechanisms involved in selective visual attention. In the current study, besides the evoked P300 component, we will analyze the ERSPs.

We hypothesize that vigilance decrement induced by prolonged engagement in a selective visual attention task impairs behavioral performance and neural activity and is evident in P300 latency and amplitude. We further hypothesize that monetary rewards improve the behavioral performance and neural activity in the low vigilance state. We apply a variant of the Eriksen Flanker Task conducted over 2 h 20 min (seven blocks) and assume that the subjects are in a lower vigilance state at the end of the task (blocks 5 and 6) than at the beginning (blocks 1 and 2). To compare the effects of motivation on performance in states of high vs. low vigilance, we introduce rewards in block 2 (during 20–40 min after task onset) and block 6 (during 100–120 min after task onset). The behavioral performance, evoked ERPs, and ERSPs were compared between high and low vigilance states with and without rewards.

## Materials and Methods

### Subjects

Twenty healthy participants (eight males), ranging from 18 to 28 [mean = 21.9, standard deviation (SD) = 2.4] years of age, were recruited from the university population. Participants reported that they had no history of smoking, sleep problems, or use of prescription medication. None worked the night shift. Furthermore, they all had normal or corrected-to-normal visual acuity, and they were right-handed according to their own report. The participants were compensated for their participation. The study was conducted in accordance with the Declaration of Helsinki and was approved by the ethics committee of Liaoning Normal University. Informed consent was obtained from each subject prior to the study.

### Measures

#### Task and Stimuli

A version of the Eriksen Flanker Task (Eriksen and Eriksen, 1974) was adopted. A five-letter string stimuli with a central target letter (M/N) and four-remaining flanker letters (N/M) were used. The letters M and N were more similar with increased complexity in comparison to the original version with the letters H and S (Gulbinaite et al., 2014). In congruent trials (MMMMM or NNNNN), the target letter (the middle letter in the five-letter string) was identical to the flankers, whereas in incongruent trials (MMNMM or NNMNN), the target letter differed from the flankers. The participants were instructed to press the left button with the left index finger if the target was M and the right button with the right index finger if the target was N as soon as possible while maintaining a high level of accuracy.

All stimuli were presented as white against a black background on a computer screen. At the beginning of the task, there was a fixation cross in the center of the screen (0.32° × 0.32°). Each letter of the string had a height and width of 0.24° visual angle. The letters were 0.05° apart to increase the error rates (Boksem et al., 2008). After 1,000 ms, the fixation cross was replaced by the five-letter string. The stimuli disappeared after 200 ms and—for the subjects to provide the response—were followed by a time interval, which elapsed until the response button was pressed or until 600 ms. An additional 200-ms interval was provided for the subjects to realize a possible erroneous response. Finally, the feedback indicating task performance was presented for 1,000–1,500 ms, depending on the response time. Feedback was presented with given responses (“Correct,” “Error,” or “Miss”) at a width of 0.5 cm. Each trial lasted 3 s in total. The trial structures are depicted in Figure 1. Congruent (60%) and incongruent (40%) trials were presented in random order (Tops et al., 2006).

#### Reward

Although individuals present differences in sensitivity to reward, the monetary reward has been corroborated to be an effective means of motivation manipulation (Paschke et al., 2015). Participants were told that in one or some blocks, for each correct response, they would receive bonus money, and they would not lose money for errors or misses. Participants could earn up to 100 RMB (approximately 12.8€) in addition to a basic sum of 50 RMB (approximately 6.4€) payment. The amount of money was evaluated proportionally to students’ monthly expenses when manipulating motivation. To maintain the effectiveness of the reward, it was stressed that they would receive the bonus if the average accuracy of the reward blocks was more than 90%; otherwise, they would lose it. For the feedback in the reward blocks, the correct responses “Correct” coupled with “+ RMB” were 1 cm in width, and the “Error” or “Miss” responses were similar to the nonreward blocks (Figure 1).

### Procedure

The participants were informed that they should abstain from alcohol, tea, and coffee for 24 h before the experiment. After arriving at the laboratory, they were given the written task instructions. They were asked to leave their watches and mobile phones outside the laboratory so that they had no indication of time during the measurement. The participants were then seated in front of a 19-inch PC monitor (1,280 × 1,024 pixels) at a distance of 0.9 m in a dimly lit, sound-attenuated, and electrically shielded room. Participants practiced the task before the formal experiment day to achieve an accuracy of 90% (those with an accuracy of <90% were not included in this study). Moreover, the reward was introduced in the practice experiment to build the association between task performance and monetary reward already prior to the experiment to avoid different time of reward exposure in high vs. low vigilance states in the formal experiment. On the experiment day, prior to the start of the formal experiment, participants performed the task for 10 min (200 trials) to adapt to the task. In the formal experiment, they were instructed to respond to the target letter presented in seven blocks of 20 min, for a total of 2 h 20 min (2,800 trials). Among the seven blocks, the monetary reward was introduced in blocks 2 and 6. The procedures can be found in Figure 1. The task blocks 1–4 were performed to induce vigilance decrement. To avoid any anticipatory effect of experiment ending, the additional no-reward block 7 was performed after the rewarded block 6. There was no rest during the experiment or any subjective questionnaires to maintain task performance and avoid the effects of short breaks alleviating fatigue. Prior studies have shown that even short breaks can increase task performance, making it difficult to evaluate whether the performance recovery results from motivation or the short break (Helton and Russell, 2015; Lim and Kwok, 2016). To maintain task performance, subjects were asked to focus their attention on the target letter presented in the center of the screen. The subjects were informed of the beginning and end of the reward blocks by instructions displayed on the screen. At the end of the task, the average accuracy of reward blocks was calculated to determine whether they would receive the bonus money or not.

### EEG Recording and Processing

The EEGs were recorded using 64 Ag/AgCl electrodes attached to an electro cap according to the International 10-20 System. An ANT Neuro EEG amplifier was used to record EEG signals sampled at a digitization rate of 500 Hz. Horizontal and vertical electrooculograms were recorded from the outer canthi of the eyes and above and below the left eye. The electrode impedance was kept below 10 kΩ, and the EEG was online referenced to the CPz channel.

In the offline analysis, EEG data were notch filtered at 50 Hz. Next, a digital high-pass filter of 0.5 Hz and a low-pass filter of 30 Hz were applied. After removing the direct current (DC) component, the EEG signals were denoised using the wavelet threshold method (Zhang et al., 2018), wherein the wavelet coefficient threshold was set to *abs* (mean ± 3 × SD). If the absolute value of the wavelet coefficients exceeded the threshold, the coefficients were reset to one-quarter of the average value. The data were re-referenced to the average of the mastoid references (M1, M2). The ERP epochs from 200 ms before to 800 ms after stimulus onset were extracted. Finally, by using the Icasso software (Himberg and Hyvärinen, 2003), independent artifact components (e.g., blinks, movements, etc.) were removed through visual inspection.

### Data Analysis

To study the effects of the reward state (i.e., no-reward vs. reward) on the behavioral and electrophysiological measures in the states of high vs. low vigilance, four blocks (blocks 1, 2, 5, and 6) were selected. The subjects were provided with monetary rewards in blocks 2 and 6. In both high (blocks 1 and 2) and low (blocks 5 and 6) vigilance states, the reward blocks were introduced after the no-reward blocks. In summary, the analysis was based on 2 × 2 comparisons, representing the no-reward high vigilance (NRHV) condition in block 1, reward high vigilance (RHV) condition in block 2, no-reward low vigilance (NRLV) condition in block 5, and reward low vigilance (RLV) condition in block 6.

#### Behavioral Performance

For each participant, the accuracy, mean RT, and number of omitted responses were calculated. Only responses occurring between 100 and 600 ms were included in the RT analysis. A response time equal to zero was regarded as an omitted response. The accuracy was calculated as the percentage of correct responses in each block. We addressed the main effects and interactions of the vigilance state and the reward state on task performance. In addition, the effect of congruency (congruent vs. incongruent) was also tested for accuracy, RT, and omitted responses.

#### Event-Related Potentials

ERPs were analyzed with MATLAB 2015b. First, the individual correct trials whose amplitude was out of range (max >75 μv, baseline max >30 μv) were rejected, and then the baseline 200 ms before stimulus onset was subtracted from the waveforms. Next, trials were averaged across blocks for each subject. The mean (with SD in parentheses) number of trials across all subjects for NRHV, RHV, NRLV, and RLV were 236 (82), 232 (65), 234 (64), and 238 (64), respectively. The P300 amplitude and latency were quantified for further analysis. Based on some earlier studies (Polich and Kok, 1995; Kuba et al., 2012; van Dinteren et al., 2014) and topographic activations in our study, eight electrodes (FC1, FC2, FCz, C1, C2, Cz, CP1, and CP2) were chosen for the P300 analysis. A time window of 440–660 ms for the P300 component was selected. The P300 latency values were calculated as the time of maximum amplitude within the time window of the P300 component (Luck, 2005).

#### EEG Spectra

The EEG spectral power was assessed by calculating the ERSP using the continuous wavelet transform (CWT; Zhang et al., 2018). The complex Morlet wavelet was adopted for the CWT analysis, by which the time-dependent signals were evaluated at each sampling instant with a central frequency band of 1.5 Hz covering frequencies from 1 to 30 Hz, with a frequency step of 0.5 Hz. Additionally, we normalized the power spectra with the subtraction change from −1,000- to 0-ms baseline. For quantifying the oscillatory dynamics, we focused on separate time windows in the analysis of two frequency bands (Figure 5). According to the maximum power of the different frequency bands, statistical analysis was performed within the time window of 440–660 ms for the delta band (1–4 Hz) and within the time window of 300–600 ms for the theta band (4–8 Hz). In order to account for the effect of phase-locked (evoked response) activity in the induced oscillations, we also analyzed the induced activations by subtracting the averaged evoked response from each epoch prior to the wavelet analysis. The results of this analysis are provided in the Supplementary Materials.

**Figure 2:**
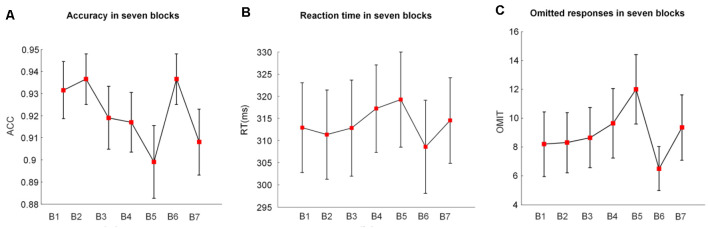
The mean values and standard deviations (SDs)/sqrt (subjects) of **(A)** the accuracy (ACC), **(B)** the reaction time (RT), and **(C)** the omitted responses (OMIT) in seven blocks. B indicates block (e.g., B1 is block1); B2 and B6 were introduced with monetary rewards.

**Figure 3:**
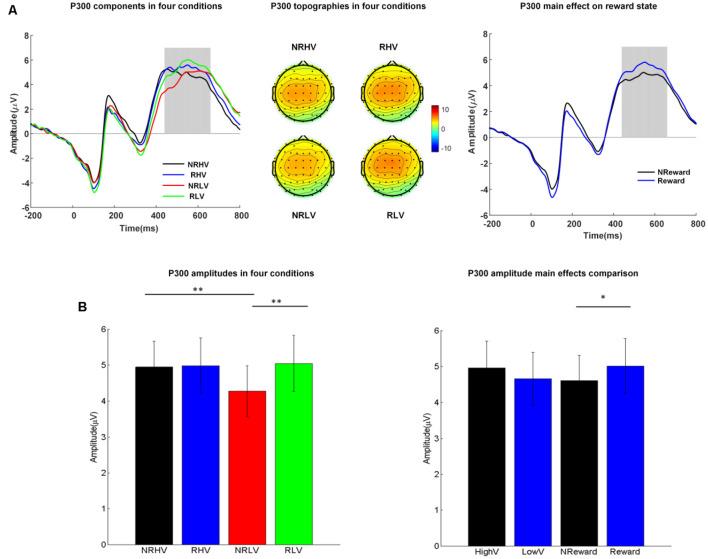
**(A)** The P300 waveforms (left) averaged from electrodes of FC1, FC2, FCz, C1, C2, Cz, CP1, and CP2, the topographies (middle) in the four conditions, and P300 waveforms coalesced for the reward vs. non-reward states (right). **(B)** Mean values and standard error of the P300 amplitude in the four conditions (left) and in the two main factors (right). HighV (high vigilance blocks) = (NRHV + RHV)/2, LowV (low vigilance blocks) = (NRLV + RLV)/2, NReward (no-reward blocks) = (NRHV + NRLV)/2, and reward (reward blocks) = (RHV + RLV)/2. Analysis of variance (ANOVA) results were marked by *p < 0.05 and **p < 0.01.

**Figure 4:**
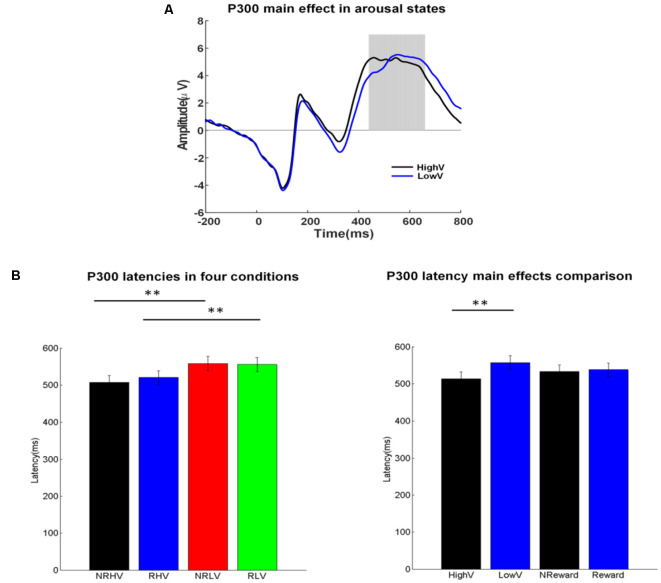
**(A)** P300 waveforms coalesced for the high vs. low vigilance states. **(B)** Mean values and standard error for the P300 latency in the four conditions (left) and for the two main factors (right). The ANOVA results were marked by *p < 0.05 and **p < 0.01.

**Figure 5:**
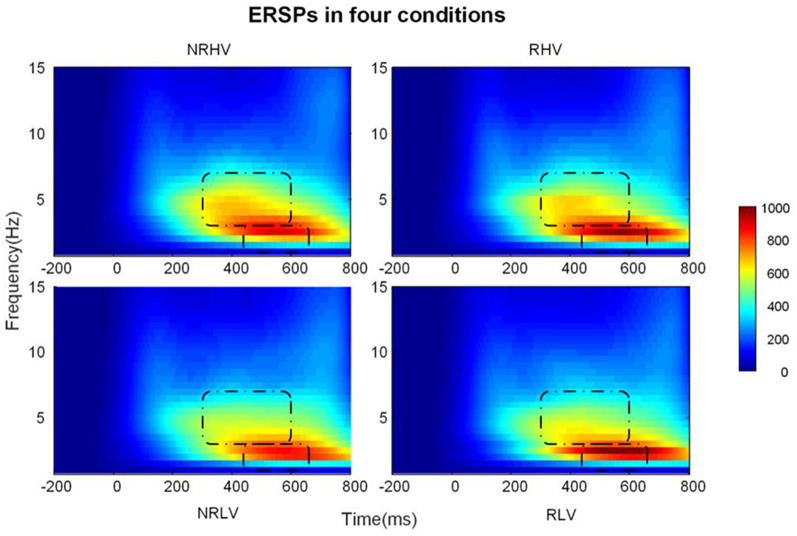
The time-frequency representations in the four conditions averaged over subjects.

#### Statistical Analysis

Data were analyzed using the IBM SPSS software (version 22.0), Chicago: SPSS Inc. The significance level *p* < 0.05 was used, and all results were reported under the 2-tailed condition. One-way repeated-measures analysis of variance (ANOVA) with the blocks 1, 3, 4, and 5 was used to test the hypothesis that behavioral performance deteriorates with time-on-task. Blocks 2 and 6 with an additional influence of motivation and block 7 with an effect of approaching the end of the task were excluded to capture the changes purely due to time-on-task. Moreover, behavioral, time domain, and time-frequency domain data were subjected to 2 × 2 [vigilance states (high and low) × reward states (no-reward and reward)] repeated-measures ANOVA. In case of significant interaction and/or main effects, a follow-up ANOVA was applied to separately test the effect of the vigilance state in no-reward and reward conditions (NRLV vs. NRHV indicates the effects of vigilance decrement) and the effect of reward in low and high vigilance states (RHV vs. NRHV and RLV vs. NRLV indicate the effects of motivation in the high and low vigilance states, respectively). The Greenhouse–Geisser correction was used as the adjusted report, and the effect size was determined using adjusted partial *η*^2^ ($\eta_{\text{ap}}^{2}$; Mordkoff, 2019).

The effect of congruency was initially tested with 2 × 2 × 2 ANOVA (congruency, vigilance state, and reward state). However, as no interaction was found for congruency, the effects of the reward and vigilance states were tested with congruent and incongruent trials integrated together. The correlations between performance (accuracy, RT, and omitted response) and ERPs (P300 amplitude and latency) were calculated using the Pearson Correlation Coefficient to study the association between the behavioral and electrophysiological measures in different vigilance and reward states.

## Results

### Behavioral Performance

Figure 2 illustrates the alterations of behavioral performance (accuracy, RT, and number of omitted responses) with time-on-task. Based on the one-way repeated-measures ANOVA, we found that the accuracy significantly decreased (*F*_(1.37,25.93)_ = 4.44, *p* = 0.02, $\eta_{\text{ap}}^{2}$ = 0.15) with time-on-task. Meanwhile, the RT (*F*_(2.37,44.99)_ = 3.97, *p* = 0.03, $\eta_{\text{ap}}^{2}$ = 0.13) and the number of omitted responses (*F*_(2.55,48.45)_ = 4.12, *p* = 0.02, $\eta_{\text{ap}}^{2}$ = 0.14) significantly increased along with the prolonged task performance.

#### Accuracy

In the 2 (vigilance states) × 2 (reward states) ANOVA analysis, there was a significant main effect of the reward state (*F*_(1,19)_ = 6.02, *p* = 0.03, $\eta_{\text{ap}}^{2}$ = 0.21 and a significant vigilance state × reward state interaction (*F*_(1,19)_ = 7.38, *p* = 0.01, $\eta_{\text{ap}}^{2}$ = 0.24). When the vigilance states were contrasted separately for reward and no-reward conditions, the accuracy was lower in the low vigilance state than in the high vigilance state in the no-reward condition (NRLV: mean = 0.88, *SD* = 0.12, NRHV: mean = 0.93, *SD* = 0.06, *F*_(1,19)_ = 5.24, *p* = 0.03, $\eta_{\text{ap}}^{2}$ = 0.17). There was no difference between the rewarded low and high vigilance states (RLV: mean = 0.94, *SD* = 0.05, RHV: mean = 0.94, *SD* = 0.05, *F*_(1,19)_ = 0.00, *p* = 1.00, $\eta_{\text{ap}}^{2}$ = 0.00). The monetary reward played a role only in the low vigilance state. The accuracy was higher in the rewarded than in the no-rewarded low vigilance condition (*F*_(1,19)_ = 7.37, *p* = 0.01, $\eta_{\text{ap}}^{2}$ = 0.24), although there was no significant difference between the rewarded and the no-rewarded high vigilance conditions (*F*_(1,19)_ = 0.47, *p* = 0.50, $\eta_{\text{ap}}^{2}$ = −0.03).

#### Reaction Time

There was a significant main effect of the reward state on the RT (*F*_(1,19)_ = 10.95, *p* < 0.01, $\eta_{\text{ap}}^{2}$ = 0.33). The follow-up ANOVA indicated that the RT increased with vigilance decrement in the no-reward condition (NRLV: mean = 319.22, *SD* = 49.15, NRHV: mean = 311.02, *SD* = 46.42, *F*_(1,19)_ = 5.52, *p* = 0.03, $\eta_{\text{ap}}^{2}$ = 0.18). There was no significant difference between the low and high vigilance states in the reward condition (RLV: mean = 308.58, *SD* = 45.25, RHV: mean = 309.83, *SD* = 47.25, *F*_(1,19)_ = 0.21, *p* = 0.65, $\eta_{\text{ap}}^{2}$ = −0.04). When rewards were provided in the states of low and high vigilance, the RT was faster in the low vigilance state (*F*_(1,19)_ = 8.38, *p* = 0.01, $\eta_{\text{ap}}^{2}$ = 0.27) but was not improved in the high vigilance state (*F*_(1,19)_ = 1.75, *p* = 0.20, $\eta_{\text{ap}}^{2}$ = 0.04).

#### Omitted Responses

There was a significant main effect of the reward state on the number of omitted responses (*F*_(1,19)_ = 9.22, *p* = 0.01, *η*^2^ = 0.29). The follow-up ANOVA revealed that the omitted responses increased with the decrease in vigilance in the no-reward condition (*F*_(1,19)_ = 5.39, *p* = 0.03, $\eta_{\text{ap}}^{2}$ = 0.18). No difference was found between low and high vigilance states in the reward condition (*F*_(1,19)_ = 0.07, *p* = 0.80, $\eta_{\text{ap}}^{2}$ = −0.05). The number of omitted responses decreased in the state of low vigilance (*F*_(1,19)_ = 10.94, *p* = 0.01, $\eta_{\text{ap}}^{2}$ = 0.33) after motivation manipulation, although it did not change in the state of high vigilance (*F*_(1,19)_ = 1.97, *p* = 0.18, $\eta_{\text{ap}}^{2}$ = 0.05).

#### Congruency

Regarding the congruency (congruent × incongruent), we found significant main effects of congruency on accuracy (*F*_(1,19)_ = 18.07, *p* < 0.01, $\eta_{\text{ap}}^{2}$ = 0.46), RT (*F*_(1,19)_ = 32.75, *p* < 0.01, $\eta_{\text{ap}}^{2}$ = 0.61), and omitted responses (*F*_(1,19)_ = 9.65, *p* = 0.01, $\eta_{\text{ap}}^{2}$ = 0.30). The congruent condition showed a higher accuracy (congruent: mean = 0.94, *SD* = 0.01, incongruent: mean = 0.91, *SD* = 0.02), faster RTs (congruent: mean = 306.93, *SD* = 10.08, incongruent: mean = 315.28, *SD* = 9.84), and less omitted responses (congruent: mean = 2.98, *SD* = 0.69, incongruent: mean = 5.45, *SD* = 1.31) than the incongruent condition. However, no significant interaction between congruency and vigilance state or between congruency and reward state was observed in behavioral performance.

### Event-Related Potentials

#### P300 Components

##### Amplitude

The left part of Figure 3A shows the averaged ERP amplitude waveforms with the time window of interest (P300 response at 440–660 ms after stimulus onset) depicted by a gray rectangle. The middle part of Figure 3A shows the corresponding topographies in the four experimental conditions, whereas Figure 3B illustrates the differences in P300 amplitude between the four conditions (left) and between the two main factors of vigilance state and reward state (right).

The repeated-measures ANOVA showed a significant main effect of the reward state on the P300 amplitude (*F*_(1,19)_ = 7.08, *p* = 0.02, $\eta_{\text{ap}}^{2}$ = 0.23) and a significant interaction between the vigilance state and reward state (*F*_(1,19)_ = 6.78, *p* = 0.02, $\eta_{\text{ap}}^{2}$ = 0.22). The follow-up ANOVA revealed that the P300 amplitude decreased with vigilance decrement (NRLV: mean = 4.17, *SD* = 2.68, NRHV: mean = 4.81, *SD* = 2.74, *F*_(1,19)_ = 9.99, *p* = 0.01, $\eta_{\text{ap}}^{2}$ = 0.31) in the no-reward condition, although no significant difference was found between the low and high vigilance states in the reward condition (RLV: mean = 4.96, *SD* = 3.01, RHV: mean = 4.89, *SD* = 2.98, *F*_(1,19)_ = 0.07, *p* = 0.80, $\eta_{\text{ap}}^{2}$ = −0.05). When the effect of reward was tested in states of low and high vigilance separately, the reward improvement presented only in the low vigilance state (*F*_(1,19)_ = 15.88, *p* < 0.01, $\eta_{\text{ap}}^{2}$ = 0.43) and not in the high vigilance state (*F*_(1,19)_ = 0.12, *p* = 0.74, $\eta_{\text{ap}}^{2}$ = −0.05).

Regarding the congruency (congruent vs. incongruent), a significant main effect of congruency was found for the P300 amplitude (*F*_(1,19)_ = 22.19, *p* < 0.01, $\eta_{\text{ap}}^{2}$ = 0.51). The amplitude was higher in the congruent condition (mean = 5.07, *SD* = 1.15) than in the incongruent condition (mean = 4.35, *SD* = 1.14). No interaction from congruency × vigilance state or congruency × reward state was detected.

##### Latency

Figure 4A illustrates the ERP waveforms in high and low vigilance states (reward and nonreward blocks coalesced), and Figure 4B shows the differences in P300 latency in the four experimental conditions (left) and the two main factors of vigilance state and reward state (right).

There was a significant main effect of the vigilance state on the P300 latency (*F*_(1,19)_ = 52.20, *p* < 0.01, $\eta_{\text{ap}}^{2}$ = 0.72) and an interaction between the vigilance state and the reward state (*F*_(1,19)_ = 6.55, *p* = 0.02, $\eta_{\text{ap}}^{2}$ = 0.22). Separate ANOVAs revealed the clear effects of vigilance states regardless of rewards. The P300 latency increased in the low vigilance state compared with the high vigilance state in both the no-reward (NRLV: mean = 557.45, *SD* = 84.71, NRHV: mean = 506.85, *SD* = 81.31, *F*_(1,19)_ = 45.52, *p* < 0.01, $\eta_{\text{ap}}^{2}$ = 0.69) and reward conditions (RLV: mean = 554.65, *SD* = 83.94, RHV: mean = 519.55, *SD* = 80.44, *F*_(1,19)_ = 37.97, *p* < 0.01, $\eta_{\text{ap}}^{2}$ = 0.65). When the effect of reward was tested in the states of low and high vigilance, there was no difference between reward and no-reward conditions in the low vigilance state (*F*_(1,19)_ = 0.37, *p* = 0.55, $\eta_{\text{ap}}^{2}$ = −0.03), but we did find a decrease in the high vigilance state (*F*_(1,19)_ = 6.81, *p* = 0.02, $\eta_{\text{ap}}^{2}$ = 0.23) after manipulating motivation.

Regarding congruency (congruent vs. incongruent), a significant main effect was found on the P300 latency (*F*_(1,19)_ = 8.91, *p* = 0.01, $\eta_{\text{ap}}^{2}$ = 0.28). The latency was shorter in the congruent (mean = 465.59, *SD* = 8.43) than in the incongruent conditions (mean = 471.48, *SD* = 9.41). For P300 latency, no interaction from congruency × vigilance state or from congruency × reward state was detected.

#### Correlations Between ERPs and Behavioral Performance

To investigate the associations between task performance and ERPs affected by motivation and vigilance states, the correlations between the behavioral measures (accuracy, RT, and number of omitted responses) and ERPs (the amplitude and latency of P300) were calculated (Table 1). Significant negative correlations between the accuracy and P300 latency and significant positive correlations between the accuracy and P300 amplitude were detected. Additionally, the number of omitted responses and the RT were negatively correlated with the P300 amplitude and positively correlated with the P300 latency. Scatter diagrams showing the relationships between the behavioral measures and P300 amplitude and latency can be found in Supplementary Figure S1.

**Table 1:** Correlations between behavioral performance and P300 measures in the four conditions.

	NRHV	RHV	NRLV	RLV
Model	*r*	*r*	*r*	*r*
ACC vs. AMP	0.40	0.57*	0.60*	0.59*
ACC vs. LAN	−0.52*	−0.53*	−0.53*	−0.41
RT vs. AMP	−0.65**	−0.68**	−0.82**	−0.83**
RT vs. LAN	0.54*	0.51*	0.53*	0.64*
OMIT vs. AMP	−0.39	−0.45*	−0.65**	−0.65**
OMIT vs. LAN	0.54*	0.46*	0.71**	0.59*

Note: The AMP and LAN represent the amplitude and latency of P300. ACC and OMIT represent the accuracy and number of omitted responses. Correlation cofficients in 2-tailed condition were marked by *p < 0.05 and **p < 0.01. The correlations were corrected by executing the Benjamini and Hochberg procedure for controlling the false discovery rate (FDR; Benjamini and Hochberg, 1995).

### ERSP Analysis

**Figure 6:**
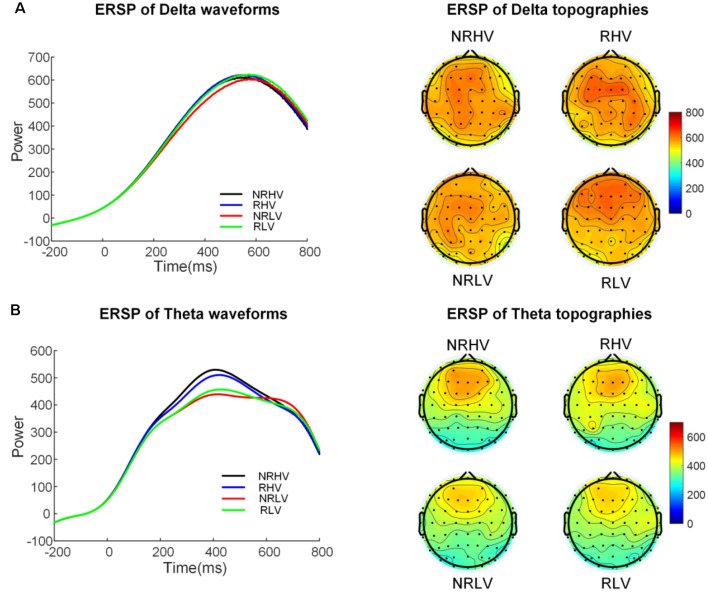
The temporal waveforms of power modulation and topographies of the delta **(A)** and the theta band **(B)** in the four experimental conditions.

Figure 5 illustrates the time-frequency representations (averaged over electrodes F1, F2, Fz, FC1, FC2, FCz, C1, C2, Cz, CP1, and CP2) in the four experimental conditions. A clear modulation of frequencies of approximately 1–4 Hz is visible in the time window of 440–660 ms. Separable modulations of approximately 4–8 Hz (in the time window of 300–500 ms) appear visually earlier than 1–4 Hz over the four conditions. The corresponding frequency bands and time windows are indicated by the dotted-line boxes. We also calculated the induced time-frequency representations after removing the phase-locked evoked responses from the total power (Supplementary Figure S2). Figure 6A illustrates the topographic distribution (right) and power waveforms (left) averaged across the electrodes (referred above) corresponding to the delta band (averaged over 1–4 Hz). Figure 6B draws the topographic distribution (right) and power waveforms (left) of the theta band (averaged over 4–8 Hz), with activations in the frontal electrodes (F1, F2, Fz, FC1, FC2, and FCz).

In the 2 (vigilance states) × 2 (reward states) ANOVAs, for the delta band power, we found a significant interaction between the vigilance state and reward state (*F*_(1,19)_ = 7.28, *p* = 0.01, $\eta_{\text{ap}}^{2}$ = 0.24). When the effect of the vigilance state was tested in the no-reward and reward conditions, the delta band power decreased with vigilance decrement in the no-reward condition (NRLV: mean = 703.43, *SD* = 162.89, NRHV: mean = 768.16, *SD* = 153.49, *F*_(1,19)_ = 8.72, *p* = 0.01, $\eta_{\text{ap}}^{2}$ = 0.28), but no significant difference was detected between low and high vigilance states in the reward condition (RLV: mean = 750.86, *SD* = 206.30, RHV: mean = 757.08, *SD* = 200.46, *F*_(1,19)_ = 0.05, *p* = 0.82, $\eta_{\text{ap}}^{2}$ = −0.05). When the effect of reward was separately tested in the states of low and high vigilance, the effect of reward on delta band power was detected only in the low vigilance state (*F*_(1,19)_ = 4.57, *p* = 0.04, $\eta_{\text{ap}}^{2}$ = 0.19) and not in the high vigilance state (*F*_(1,19)_ = 0.23, *p* = 0.64, $\eta_{\text{ap}}^{2}$ = 0.01).

For the theta band, vigilance state had a significant main effect (*F*_(1,19)_ = 18.56, *p* < 0.01, $\eta_{\text{ap}}^{2}$ = 0.47). Follow-up ANOVA revealed that theta power was weaker in low vigilance state than in high vigilance state in both no-reward condition (NRLV: mean = 432.43, *SD* = 115.29, NRHV: mean = 512.47, *SD* = 169.54, *F*_(1,19)_ = 11.38, *p* < 0.01, $\eta_{\text{ap}}^{2}$ = 0.34) and reward condition (RLV: mean = 455.70, *SD* = 118.69, RHV: mean = 498.61, *SD* = 137.47, *F*_(1,19)_ = 6.36, *p* = 0.02, $\eta_{\text{ap}}^{2}$ = 0.21). The separate ANOVAs revealed that reward did not play a role in low vigilance state (*F*_(1,19)_ = 1.73, *p* = 0.20, $\eta_{\text{ap}}^{2}$ = 0.03) or in high vigilance state (*F*_(1,19)_ = 0.21, *p* = 0.65, $\eta_{\text{ap}}^{2}$ = −0.04).

## Discussion

We examined the alterations in behavioral performance and brain electrophysiology produced by the vigilance level and reward during a prolonged period of selective visual attention tasks. Behavioral measures (accuracy, RT, and number of omitted responses), evoked responses (P300 amplitude and latency), and spectral power (delta and theta bands) were analyzed. A clear deterioration in behavioral performance was demonstrated over time (Figure 2). The monetary reward improved the performance in accuracy, RT, and number of omitted responses only in the low vigilance state. The P300 amplitude was smaller in low than in high vigilance state; however, in the low vigilance state, reward increased the P300 amplitude to its level in the high vigilance state. The P300 latency was sensitive to vigilance decrement but insensitive to rewards, with longer latency in low than in high vigilance states. Changes in spectral power at 4–8 Hz purely reflected the vigilance level, being stronger in the high vigilance state than in the low vigilance state. Similarly, the spectral responses at 1–4 Hz also decreased with vigilance decrement. However, the reward selectively increased the spectral power at 1–4 Hz in the low vigilance state to the strength levels in the high vigilance state.

Although the time of emergence of fatigue during the prolonged performance of cognitive tasks has not been defined, earlier studies suggest cumulative effects in performance and neurophysiology with time-on-task (Boksem et al., 2005, 2006; Lorist et al., 2005; Faber et al., 2012; Möckel et al., 2015; Reteig et al., 2019). In line with these findings, our study found a significant decrease in accuracy and an increase in RT and the number of omitted responses with time-on-task (Figure 2). The decline of behavioral performance in prolonged attention tasks is in line with our assumption that time-on-task is associated with the decrement of vigilance levels. These results provided justification for testing the interactions between vigilance and reward states, where blocks 1 and 2 (first 40 min) were regarded as representing the high vigilance state, whereas blocks 5 (80–100 min) and 6 (100–120 min) were regarded as the low vigilance state. This selection was also supported by earlier findings of the effects of mental fatigue after 60- to 90-min tasks (Kastner and Ungerleider, 2000; Lorist et al., 2000; Marcora et al., 2009).

The limited processing capacity biased toward goal-directed selection is the core of selective visual attention (Robert and Duncan, 1995; Polich, 2009). The P300 component is considered as an important indicator of attentional capacity in visual tasks (Polich, 2009). In line with earlier results showing a fatigue-related decrease in P300 amplitude during brain-computer interface performance (Käthner et al., 2014), our results demonstrated that the P300 amplitude decreased with vigilance decrement during a selective visual attention task, presumably reflecting a less efficient engagement or limited capacity of attentional resources. The insufficient attention resources allocation in the low vigilance state has also been reflected by the P300 latency, which is thought to provide a specific index for the timing of information processing and stimulus evaluation (Polich and Kok, 1995; Verleger, 1997; Käthner et al., 2014). In our study, the P300 latency was significantly prolonged in the state of low vigilance, in line with earlier studies (Kutas et al., 1977; Boksem et al., 2006; Kato et al., 2009). The result indicates that prolonged task performance accompanies longer evaluation time for processing information. Therefore, in agreement with existing studies, our results demonstrated a decrease in P300 amplitude and an increase in latency along with vigilance decrement.

The close link between the modulations in behavioral performance and the changes in brain electrophysiology is demonstrated by strong correlations between the behavioral and P300 measures (Table 1). It is noteworthy that, although the RT correlates with both P300 amplitude and latency, the association between the decrease of P300 amplitude and the increase in RT in particular is clear (Supplementary Figure S1).

After reward manipulation, the P300 amplitude increased only in the low vigilance state, reaching the same level as in the high vigilance state. Our results are consistent with earlier studies demonstrating a monetary-reward based improvement of neural measures in mental fatigue (Boksem et al., 2006; Hopstaken et al., 2015). We further provide quantitative evidence for the recovery of attentional resources from low vigilance to high vigilance states. Our results verify that, when rewards are provided, the capacity of attentional resources in the low vigilance state can reach the level of capacity in the high vigilance state, at least within the limited time duration of 2 h 20 min for a visual attention task.

Interestingly, there was no significant reward-induced improvement in P300 latency either in high or low vigilance states despite the reward-related improvement in RT in the low vigilance state. These results are in line with a previous study (Boksem et al., 2006), suggesting that the P300 latency is an unstable electrophysiological marker of motivation compared with the P300 amplitude. These diverging results concerning the RT and P300 latency could be interpreted to indicate improvement in motor response generation (as reflected by the RT) but not in the preceding stage of information processing (as reflected by the P300 latency). However, these results might also reflect the complex composition (subcomponents) of the P300 responses. The P300 component has been shown to comprise two subcomponents—P3a and P3b—with different functional correlations (Demiralp et al., 2001). The P3a with frontal topography has been suggested to contribute to attention engagement in top-down task-relevant processing, whereas the P3b with centroparietal topography has been linked to the level of cognitive workload and memory encoding (Polich, 2009). They are activated in different time windows, and P3a usually emerges earlier than P3b (Polich, 2009). Although we fail to disentangle the two subcomponents in this study, it is possible that, in addition to changes in the amplitude, also changes in the emphasis of these neural subprocesses are associated with vigilance decrement. This complicates the interpretation of latency measures.

The topography of the P300 component in the present study is more anterior than that in some earlier studies (Demiralp et al., 2001; Käthner et al., 2014). This likely reflects the task requirements of the present study. To successfully perform a selective visual attention task, humans are able to filter out task-irrelevant stimuli and engage their limited capacity in task-relevant processing (Robert and Duncan, 1995). Our task is likely to harness—although it was not designed to differentiate—these two subprocesses. The modified Eriksen Flanker Task applied in our study required responses for every trial and was specifically adapted to make the target letter distinction visually hard, emphasizing the need for the active inhibition of the flankers. Some interpretations, especially in studies showing anteriorly located P300 generators, emphasize the role of the inhibitory control underlying the modulation of P300 responses, for example, as a result of aging (Kuba et al., 2012; van Dinteren et al., 2014). Importantly for the present findings, this interpretation is also sensible in the context of vigilance decrement, which is often accompanied by a reduction in the capacity for top-down inhibitory control (Guo et al., 2018).

P300 seems to provide a reliable measure of cognitive performance, but the analysis of phase-locked ERP offers only limited windows to explore the underlying neural processes in more detail. Previous studies have indicated that both delta (Keller et al., 2017) and frontal theta oscillations (Knyazev, 2012) are involved in visual attention tasks, although the influence of time-on-task was not studied in these studies. Focusing on the spectral patterns and the power modulations at different frequency bands may provide additional sensitivity to separate vigilance- and reward-related processes in the brain. The oscillatory activity at low-frequency bands [i.e., the delta (1–4 Hz) and theta (4–8 Hz) bands] has been shown to increase during the transition to the low vigilance state in spontaneous conditions (Lal and Craig, 2001). Although the modulations of the oscillatory activity triggered by cognitive tasks are different from those from spontaneous activity, the temporal variations in the power of these frequency bands triggered by a visual attention task may tap on the same underlying processes as reported based on more spontaneous conditions.

In the present study, the changes in the spectral power at 1–4 Hz and 4–8 Hz reflected different topographies, with the delta band distributed in the centroparietal electrodes and the theta band distributed more focally in the frontal electrodes. In addition, the temporal characteristics of the changes in power in these two frequency bands differed, with an earlier emergence of the modulation at 1–4 Hz (300–600 ms) than at 4–8 Hz (440–660 ms). Therefore, it is likely that the two separable changes in spectral power reflect two distinct cognitive functions involved in selective visual attention. The theta band has been shown to be an indicator of attention allocation to task-relevant stimuli (Keller et al., 2017), whereas the delta band has been linked with internal processing in an attention task (Harmony et al., 1996). Prolonged engagement in a selective visual attention task led to the reduced spectral power in both delta and theta bands. These results are in line with the analysis of the evoked P300 responses and suggest that vigilance decrement impairs both attention allocation and information processing. In the rewarded low vigilance condition, the spectral power at approximately 1–4 Hz increased to the same level as that recorded in the high vigilance state. However, the spectral power at approximately 4–8 Hz did not increase in the rewarded low vigilance state compared to the rewarded high vigilance state. Consequently, the power in these two frequency bands was thus differently modulated by motivation. It would be tempting to associate the current findings with the distinct roles suggested for the theta and delta bands, suggesting that intrinsically driven regulation of information processing can be influenced by reward (as reflected by delta-band changes, Knyazev, 2012), but the top-down attentional control (reflected by theta-band changes, Cavanagh and Frank, 2014) is insensitive to reward. Interestingly, accumulating evidence exists linking theta oscillations with the attentional sampling of the environment, especially during higher task demands (Bastiaansen and Hagoort, 2003; Landau et al., 2015; Spyropoulos et al., 2018; Karamacoska et al., 2019). Interpreted in the context of the current results suggesting the insensitivity of the theta band to reward manipulation, theta power might subserve rather low-level attentional sampling, which is not directly linked with the reward system, at least in the context of nonprimary, extrinsic rewards such as money. On the contrary, delta band oscillations may reflect a separate compensatory mechanism (Knyazev, 2012), which supports the recovery of functions after manipulating motivation.

However, these interpretations must be treated with caution, especially regarding the role of fatigue-related oscillatory dynamics. The current experimental paradigm is not optimal for studying ongoing oscillations, and the changes in rhythmic activation are strongly linked with the visual trigger. It is important to distinguish between ongoing oscillations and stimulus-related changes in spectral power. This fact is highlighted by the detected decrease in the spectral power by the decrease in vigilance in the present study, while ongoing oscillations at low-frequency bands generally show a fatigue-related increase (Lal and Craig, 2001). When the influence of phase-locked evoked activation (Supplementary Figure S2) was removed from the spectral responses, the theta-band modulations strongly decreased. Rather than reflecting neural computation in the theta band, the time-frequency results might be at least partly driven by the phase-locked evoked responses. Our analysis can be seen as advancing the interpretability of evoked responses, and different experimental paradigms are needed to focus purely on the fatigue-related changes in oscillatory dynamics.

Based on our results, vigilance decrement changes the neural processes underlying selective visual attention, as demonstrated by the changes in the spectral power at 1–4 Hz and at 4–8 Hz, as well as evoked P300 response. Motivation plays a different role in the high and low vigilance states, with improvement of performance only in the low vigilance state. This appears inconsistent with the active fatigue framework (which states that the vigilance decrement is the result of the depletion of cognitive resources, and motivation cannot improve the performance impaired by vigilance decrement, Helton and Warm, 2008) because the impairment in the state of low vigilance is improved after motivation manipulation. On the other hand, our results seem to agree with the motivation control framework—that vigilance decrement is a subconscious balancing between the costs and benefits to expend or conserve energy (Kurzban et al., 2013a). When the cost of efforts to carry out a task outweighs the benefits, humans are unwilling to do so, leading to vigilance decrement. However, not all of the neural measures are improved after providing reward. The P300 latency in the low vigilance state was not modulated by reward. Furthermore, the spectral power in the delta but not in the theta band was modulated by motivation manipulation, which means that motivation partially alleviates neural activity in the low vigilance state. In general, our results imply that motivation is not enough to completely restore the impairment induced by vigilance decrement and provide support for the mental fatigue framework, which integrates the evaluation of expected rewards and energetic costs (Boksem and Tops, 2008).

Further studies are inevitably needed to establish a more comprehensive picture of the underlying neural processes affected by motivation and vigilance states. We only analyze the changes in high vs. low vigilance states; nevertheless, focusing on the ongoing changes while performing the task can significantly advance the understanding of the dynamic emergence of mental fatigue. Our study did not consider the effects of monetary values during a long period of attention task engagement. It is also not possible to completely disengage the dimensions of vigilance and motivation, as it is likely that a decrease in vigilance is also accompanied by decreased motivation to perform a task. Furthermore, providing rewards is not the only method to motivate individuals. Further studies should further elaborate the particular differences in sensitivity to reward (positive) and punishment (negative).

## Conclusion

Both the behavioral and electrophysiological measures were modulated by vigilance decrement. The neurocognitive processes were only partially recovered by manipulating rewards. In particular, increasing motivation using rewards differentially influenced brain activations in the high vs. low vigilance states, with more evident improvement in the low than in the high vigilance state. The fatigue-related decrease in latency of P300 responses did not recover with rewards, whereas the P300 amplitude increased to the same level as in the high vigilance state. The spectral power of the delta band was specifically increased by motivation, whereas the decrease of the theta band was not recovered by reward. These findings provide evidence for the dissociable effects of motivation in the states of low and high vigilance and might validate the mental fatigue framework integrating the evaluation of expected rewards and energetic costs.

## Data Availability Statement

The datasets generated and analyzed during the present study are available from the corresponding author on reasonable request.

## Ethics Statement

The studies involving human participants were reviewed and approved by Liaoning Normal University. The participants provided their written informed consent to participate in this study.

## Author Contributions

JL, HS, and TP designed the experiment. JL, CZ, and YZ analyzed the data. JL and YL collected the data. JL and TP conducted the statistics. JL, FC, TP, and TR wrote the manuscript. FC, TR, and TP provided the fundings and guidance for all the conduction of work.

## Conflict of Interest

The authors declare that the research was conducted in the absence of any commercial or financial relationships that could be construed as a potential conflict of interest.

## Supplementary Material

FIGURE S1 Scatter diagrams showing the relationships between the behavioral measures (accuracy, reaction time, and number of omitted responses) and the P300 amplitude and latency in the four conditions. NRHV is block 1 in the no-reward high vigilance state, RHV is block 2 in the reward high vigilance state, NRLV is block 5 in the no-reward low vigilance state, and RLV is block 6 in the reward low vigilance state.

FIGURE S2 Comparison of different ways of calculating time-frequency representation changes for the current data. **(A)** Calculation of the power with the continuous wavelet transform (CWT) in each trial, and then averaged (presented in the present study). **(B)** Calculation of the power with the CWT from epochs, from which the contribution of averaged evoked responses are removed from each trial (averaged ERP is subtracted from each trial). **(C)** Calculation of the power with the CWT from epochs averaged in the evoked responses (spectra power of averaged ERP).
