# Supplementary material for: Dissociable Effects of Reward on P300 and EEG Spectra Under Conditions of High vs. Low Vigilance During a Selective Visual Attention Task
Source: Front Hum Neurosci. 2020 Jun 24;14:207. doi: 10.3389/fnhum.2020.00207 (PMC7327118; doi:10.3389/fnhum.2020.00207)

FIGURE S1 | Scatter diagrams showing the relationships between the behavioral measures (accuracy, reaction time, and number of omitted responses) and the P300 amplitude and latency in the four conditions. NRHV is block 1 in the no-reward high vigilance state, RHV is block 2 in the reward high vigilance state, NRLV is block 5 in the no-reward low vigilance state, and RLV is block 6 in the reward low vigilance state.

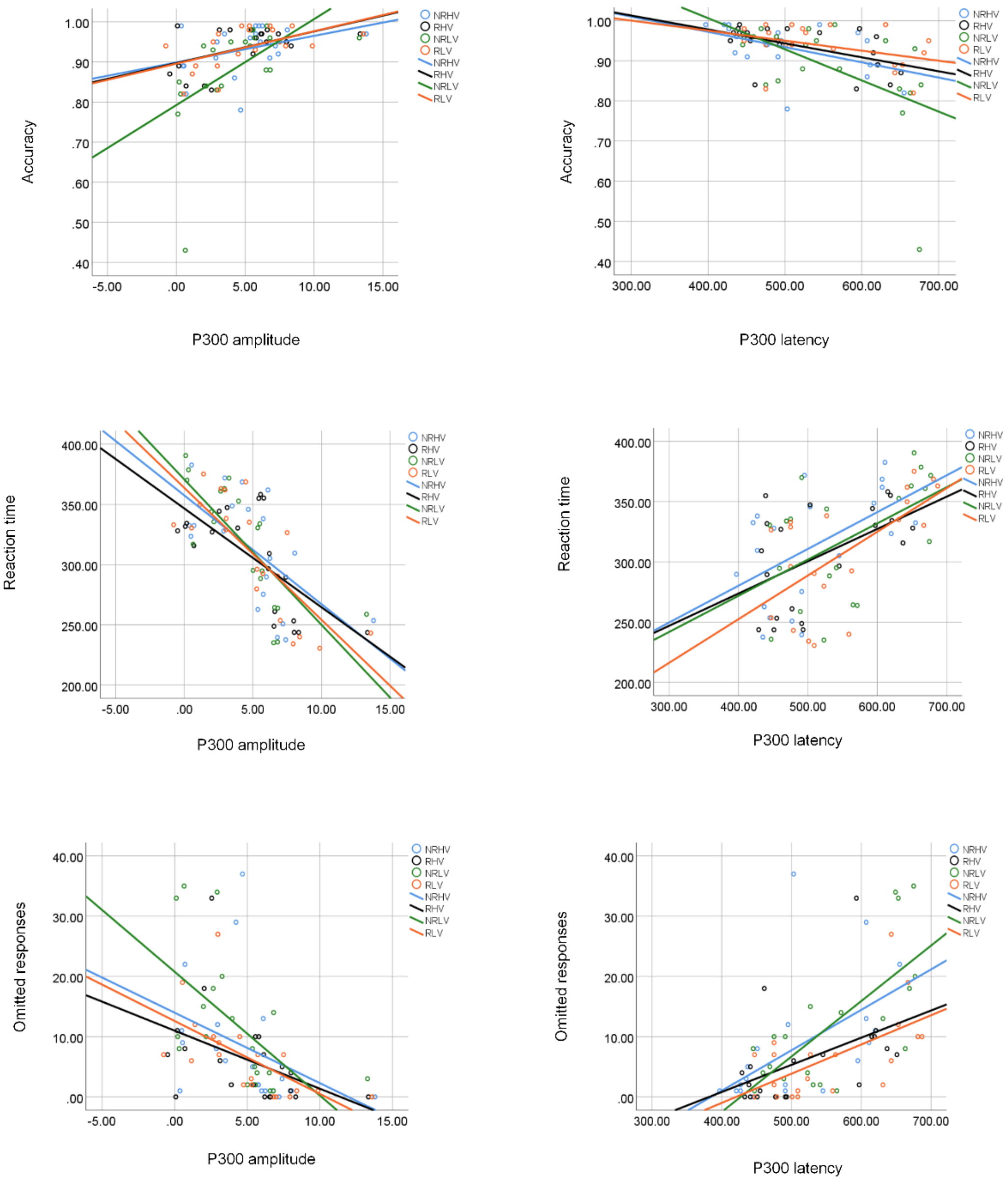

FIGURE S2 | Comparison of different ways of calculating time-frequency representation changes for the current data. (A) Calculation of the power with the continuous wavelet transform (CWT) in each trial, and then averaged (presented in the present study); (B) Calculation of the power with the CWT from epochs, from which the contribution of averaged evoked responses are removed from each trial (averaged ERP is subtracted from each trial); (C) Calculation of the power with the CWT from epochs averaged in the evoked responses (spectra power of averaged ERP).

(A)

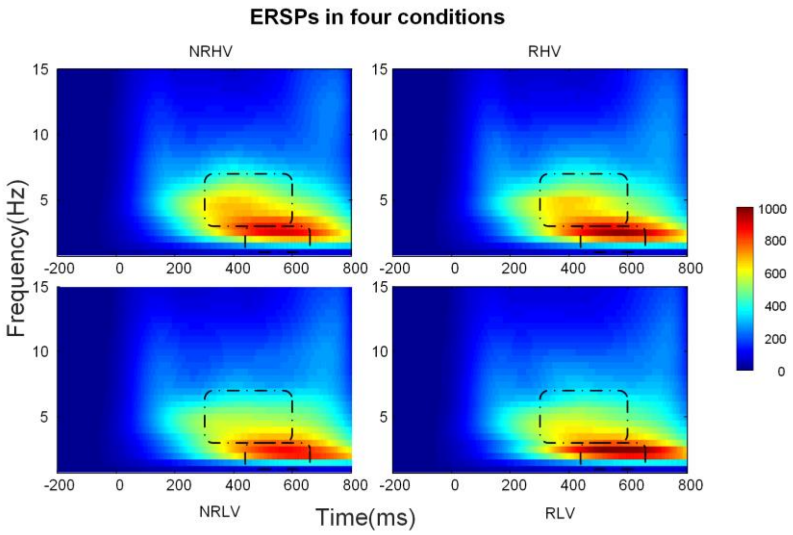

(B)

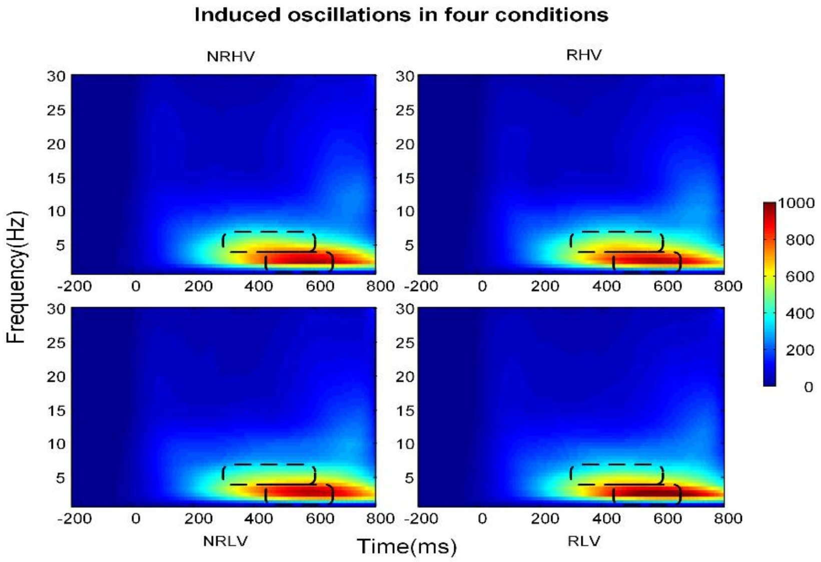

(C)

Evoked oscillations in four conditions

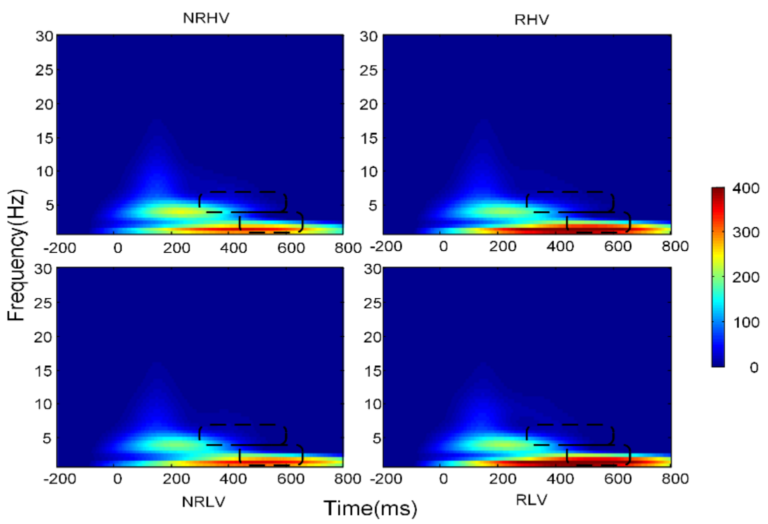

Supplement: FIGURE S1 — Scatter diagrams showing the relationships between the behavioral measures (accuracy, reaction time, and number of omitted responses) and the P300 amplitude and latency in the four conditions. NRHV is block 1 in the no-reward high vigilance state, RHV is block 2 in the reward high vigilance state, NRLV is block 5 in the no-reward low vigilance state, and RLV is block 6 in the reward low vigilance state. [file Image_1.pdf]
